# Supplementary material for: 13q Deletion Syndrome Presenting with Lymphopenia Detected Through Newborn Screening for Primary Immunodeficiencies
Source: Int J Mol Sci. 2025 Sep 23;26(19):9302. doi: 10.3390/ijms26199302 (PMC12524871; doi:10.3390/ijms26199302)
Supplement: Supplementary file 1 [file ijms-26-09302-s001.zip › ijms-3855808-supplementary.pdf]

Table S1. Chronological summary of prenatal findings by trimester in the proband with 13q deletion syndrome.

Trimester	Gestational age	Findings
**First trimester**	3–4 weeks	Threatened miscarriage; hospitalization for pregnancy preservation; maternal iron-deficiency anemia
	~12 weeks	Increased risk of preeclampsia and fetal growth restriction (FGR); multiple congenital anomalies suspected; high risk of chromosomal abnormalities; prenatal karyotyping was recommended but not performed
**Second trimester**	16–18 weeks	Maternal iron-deficiency anemia, hypothyroxinemia; suspected CNS anomalies; fetal growth restriction
	19 weeks	Confirmed FGR and multiple anomalies: CNS, congenital heart defect, urogenital malformations; high risk for chromosomal abnormalities confirmed by perinatal consultation
**Third trimester**	29 weeks	Alobar holoprosencephaly; functionally single left ventricle with tricuspid valve atresia; double outlet of the great vessels with severe pulmonary artery hypoplasia/atresia; bilateral renal hypoplasia; progressive FGR
	39 weeks	Severe growth restriction (birth weight 2050 g); alobar holoprosencephaly, congenital heart defect (single ventricle, tricuspid atresia, great vessel anomaly), renal hypoplasia; oligohydramnios; orofacial cleft suspected
